# Revisiting leader–member exchange: a review through the lens of the “too much of a good thing” effect

**DOI:** 10.3389/fpsyg.2026.1722767

**Published:** 2026-03-23

**Authors:** Kazuho Yamaura, Shima Okada, Yoko Nishihara, Naruhiro Shiozawa, Eri Mukai, Yusuke Sakaue

**Affiliations:** 1 College of Sport and Health Science, Ritsumeikan University, Shiga, Japan; 2 Institute of Advanced Research for Sport and Health Science, Ritsumeikan University, Shiga, Japan; 3 College of Science and Engineering, Ritsumeikan University, Shiga, Japan; 4 College of Information Science and Engineering, Ritsumeikan University, Osaka, Japan; 5 College of Life Sciences, Ritsumeikan University, Shiga, Japan; 6 Graduate School of Engineering Science, The University of Osaka, Osaka, Japan

**Keywords:** curvilinear relationship, emotion, intrinsic motivation, leader-member exchange (LMX), psychophysiological indicators, resource loss, too much of a good thing (TMGT)

## Abstract

**Objectives:**

This review reexamines the widely accepted belief that high-quality leader-member exchange (LMX) relationships are uniformly beneficial.

**Methods:**

Based on the “too much of a good thing” framework, this study systematically reviews new empirical evidence suggesting that excessively high LMX may yield diminishing returns and negative outcomes.

**Results:**

This review examines the unique and important contributions that high LMX relationships bring to LMX research. It highlights the emotional costs underlying non-linear patterns and presents an integrated Resource-Emotion-Motivation (REM) theoretical framework. Additionally, it discusses methodological aspects to better capture the dynamic relational process.

**Conclusion:**

Finally, based on the reviewed papers, we present the academic and practical implications for future research. This review contributes to broadening the understanding of LMX dynamics.

## 1 Introduction

Over the past several decades, leader-member exchange (LMX) theory has emerged as one of the most influential frameworks in organizational psychology. Rooted in role and social exchange theories, LMX emphasizes qualitative differences in the dyadic relationship between leaders and members (Graen and Scandura, 1987; Graen and Uhl-Bien, 1995). Early research consistently revealed that high-quality LMX relationships are associated with a broad range of positive outcomes, including improved job performance, increased job satisfaction, strengthened organizational commitment, and reduced turnover intentions (review articles: Andersen et al., 2020; Carnevale et al., 2017; Dulebohn et al., 2012; Gerstner and Day, 1997; Herman et al., 2018; Ilies et al., 2007; Kim et al., 2020; Martin et al., 2016; Mumtaz and Rowley, 2020; Rockstuhl et al., 2012; Tse et al., 2018). Consequently, both research and practice have largely embraced the normative assumption that “more is better” (i.e., higher LMX quality yields better outcomes).

However, recent empirical evidence has begun to challenge this linear assumption. The relationship between LMX and member outcomes may be more complex and potentially non-linear, rather than strictly linear. Specifically, LMX quality may yield more desirable outcomes at moderate levels, whereas excessively high LMX can lead to unintended consequences such as psychological strain and emotional exhaustion. These observations align with the theoretical framework proposed by Pierce and Aguinis (2013), termed the “too much of a good thing” (TMGT) effect. This theory asserts that even beneficial concepts exhibit diminishing returns beyond an optimal level and can become harmful. According to them, non-linear effects are not surprising phenomena but are likely quite common in management studies.

In reality, as numerous studies have shown, LMX plays a crucial role in individual members’ and workplace experiences. However, we may have treated high LMX relationships as uniformly desirable, overlooking their subtle psychological trade-offs. Therefore, this review clarifies the findings from the existing literature on the effects of LMX on TMGT. Because studies that empirically tested this phenomenon were insufficient to warrant a systematic review/meta-analysis, this review is presented as a scoping review of the existing literature.

According to Colquhoun et al. (2014), a scoping review is “a form of knowledge synthesis that addresses an exploratory research question aimed at mapping key concepts, types of evidence, and gaps in research related to a defined area or field by systematically searching, selecting, and synthesizing existing knowledge (p. 5).” Based on this definition, this paper is structured to answer the main question: Can LMX produce non-linear effects? Theoretically, we propose an explanatory framework grounded in multiple theories considered relevant to this effect. We argue that when relational benefits become high and emotional and social costs exceed available resources, the optimal relational equilibrium is disrupted, leading to the TMGT effect of LMX.

We highlight moderating factors that reveal LMX complexity and the potential psychological costs associated with it, and suggest directions for future research, including methodological perspectives. We particularly recommend measurement approaches (i.e., multimodal, longitudinal, and context-sensitive) that align research design with the subject of verification and measurement quality. This is crucial because non-linear effects are often smaller than linear effects; they can be exacerbated by issues in research design or measurement methods (Harms and Han, 2016; Krasikova et al., 2013). Finally, we present practical implications derived from the accumulated empirical research.

This paper advances LMX research in several ways. First, it organizes evidence and characteristics of the TMGT effect of LMX, providing material to reconsider the traditional “more is better” paradigm. Second, it integrates existing evidence on leadership complexity and proposes a comprehensive theoretical framework to explain this phenomenon. The new analytical perspectives suggested by this framework help elucidate potential psychological mechanisms. Third, most research in this field relies on cross-sectional survey designs, limiting the ability to capture the temporal dynamics of high LMX or its potential cognitive and emotional costs. Illustrating measurement methods for verifying the proposed framework model and presenting analytical perspectives will contribute to advancing LMX research.

### 1.1 Re-examining LMX theory from the perspective of TMGT effects

Do LMX outcomes exhibit non-linear properties? First, we attempt to explain this within the framework of LMX theory. LMX theory is grounded in role and social exchange theories. Through the role-formation process, leaders build social exchange relationships with members based on trust, obligation, and respect (Graen and Uhl-Bien, 1995). According to role theory, leaders assign important roles within an organization to members with high LMX, and these members receive support distinct from that provided to members with low LMX (Graen and Scandura, 1987). Following the principles of social exchange theory, leaders expect reciprocity for benefits received from members (Blau, 1964), and members feel obligated to assist a leader in return, adhering to the norm of reciprocity (Gouldner, 1960). Thus, members with high LMX relationships gain more resources such as support, autonomy, and information. Motivated by personal loyalty and trust toward a leader, they can leverage these resources to achieve results.

During the role-shaping process, members with high LMX experience increased opportunities to tackle complex, unstructured tasks or meet a leader’s personal demands. As the level and scope of job demands increase, a leader may reach a point at which they cannot provide members with additional resources or more attractive incentives. Members may find that job demands exceed their available psychological resources, or that a leader’s resource supply becomes insufficient. Consequently, members with excessively high LMX may become more prone to negative emotions, stress reactions, and decreased performance.

This paradox is thought to occur when the quality of LMX exceeds a threshold, and the organization’s or leader’s resources become insufficient to meet job demands. Similar concepts can be explained by the conservation of resources theory (COR: Hobfoll, 2011) and the job demands-resources (JD-R) model (Bakker and Demerouti, 2014; Bakker et al., 2023). These theories emphasize the importance of acquiring and maintaining resources, essentially arguing that greater resource availability is advantageous.

Applying this perspective, individuals can effectively manage these resources up to a certain demand level. However, when unrelenting demands and amplified obligations exceed this capacity, LMX threatens existing resource depletion and acquisition potential. Furthermore, Cognitive Load Theory (Sweller, 1988, 2011) supports this from the perspective of cognitive resources. Given the finite capacity of human cognitive processing and working memory, the complexity of tasks under high-demand conditions can lead to cognitive load exceeding an individual’s resources. This aspect likely triggers unintended effects of LMX, such as reduced learning and performance (Wu et al., 2024). Based on the above, it is anticipated that excessively high LMX can produce unintended outcomes.

## 2 Methods

This review involved a systematic literature search to identify and synthesize studies examining the TMGT effect within the LMX framework. To enhance reproducibility and transparency, a structured review procedure aligned with recommendations for reviews in organizational psychology was adopted (Baumeister and Leary, 1997; Tricco et al., 2015).

### 2.1 Search strategy and database

The literature search was conducted in November 2025 using the academic databases PsycINFO, PsycARTICLES, ProQuest, and Web of Science. Google Scholar was also used concurrently to capture an extremely broad range of literature. However, it is important to note that the low precision of this search may affect reproducibility; therefore, only articles with the search terms in their titles were included. The search terms and query were: (“leader-member exchange” OR LMX OR “leader-follower exchange” OR “leader and follower relationship”) AND (curvelinear OR non-linear OR “too much of a good thing” OR TMGT).

To ensure that records from the final survey year could be reliably identified, the search was limited to peer-reviewed academic journal articles in English published by 2024. Note that “guanxi,” a unique Chinese context, was excluded here. This is because, although guanxi and social exchange are theoretically related, they are not equivalent. Furthermore, leader-member guanxi has been shown to add additional variance beyond LMX and exhibit distinct relationship patterns (Liden et al., 2016, p. 33). Each reference was independently screened by two researchers, with their judgments blinded to one another. Eligibility was assessed based on the selection criteria. Initial screening was conducted using titles and abstracts, followed by full-text review. Disagreements were resolved through discussions between the two researchers.

### 2.2 Screening process and final selection

**FIGURE 1:**
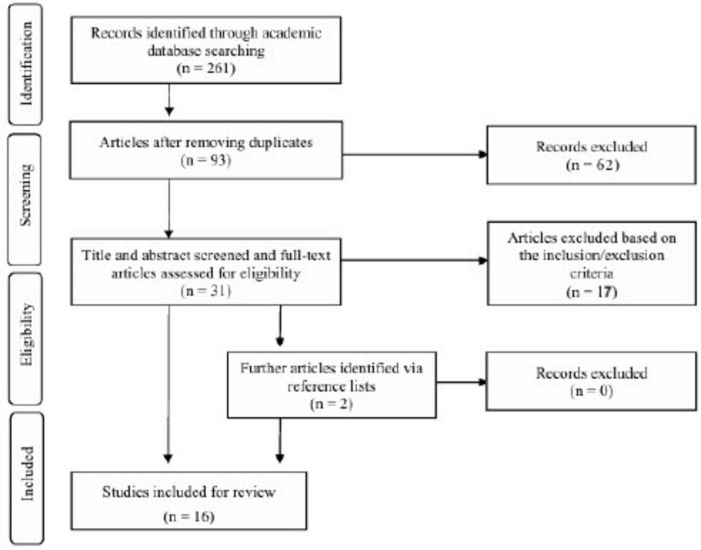
Flow diagram indicating the search procedure based on PRISMA guidelines.

After excluding duplicates, 93 articles were reviewed (Figure 1). Relevance based on titles and abstracts narrowed the selection to 31 articles. Papers were then selected using the inclusion/exclusion criteria outlined below. Studies eligible for inclusion had to meet all of the following criteria: (1) the study examined LMX as an independent variable for psychological, behavioral, or performance-related outcome variables; (2) the analysis explicitly examined non-linear or curvilinear effects (quadratic regression models, polynomial terms, and inverted U-shapes); (3) the full text was available in English; and (4) the study provided theoretical justification or a non-linear framework, or discussed relevance to related paradoxical LMX theories.

The following studies were excluded: theoretical commentaries, editorials, meta-analyses that did not include new empirical data, and studies that did not assume a curve model and focused solely on linear relationships.

Studies examining the TMGT effect of LMX differentiation were also excluded. While LMX differentiation is an important perspective (Martin et al., 2016), it is a group-level phenomenon and does not align with this study’s objective of determining whether the TMGT effect of LMX can be confirmed (Lee and Chae, 2017; Li et al., 2016; Sui et al., 2016). Furthermore, two additional papers cited within the literature were included. After the lead author thoroughly reviewed all papers, the total number of studies included in this review reached 16. Notably, three of these 16 papers each contained two separate survey studies using different samples. Therefore, the reviewed papers collectively reported analyses of 19 datasets.

## 3 Results

The selected papers were examined with respect to their theoretical frameworks, methodological designs, measurement scales, sample characteristics, and the statistical treatment of non-linear effects. Regarding statistical treatment, emphasis was placed on whether comparative analyses between linear and non-linear models were conducted. Tables 1, 2 summarize the papers included in this review.

**TABLE 1:** Articles identified for review and summaries.

References	Theoretical framework	Country	Data source	Final sample	Research design
Carnevale et al. (2020)	LMX theory; voice literature	China	Internet finance company	256 employees matched with 256 supervising managers (i.e., 256 non-nested dyads)	two-wave panel (5 weeks after Time 1)
Golden (2006)	Social exchange theory; information richness	USA	A large high-technology company located in the telecommunications industry	294 telecommuters	Cross-sectional
Harris and Kacmar (2006)	LMX theory	USA	Sample 1. State lottery Sample 2. Water management district	Sample 1. 120 employees Sample 2. 402 employees	Sample 1. cross-sectional Sample 2. cross-sectional
Harris et al. (2005)	LMX theory, motivational forces (affective, calculative, and alternative)	USA	Sample 1. Water management district Sample 2. Financial services industry	Sample 1. 402 employees Sample 2. 183 employees and 16 supervisors	Sample 1. cross-sectional Sample 2. cross-sectional
Hesselgreaves and Scholarios (2014)	LMX theory	UK	Hospitals	116 supervisor-subordinate dyads (70 with junior subordinates; 46 with senior subordinates)	Cross-sectional (multi-source, dyad design)
Hochwarter (2005)	LMX theory; affective disposition literatures	USA	Police offices	182 police officers	Cross-sectional
Ionescu and Iliescu (2021)	LMX theory; organizational justice theory	Romania	Public and private organizations, covering a wide range of sectors	274 subordinates nested under 42 leaders	Two-wave panel (3 months after Time 1)
Jian (2014)	LMX theory; social exchange theory	USA	A larger dataset collected in a survey project	235 immigrant employees working in United States	Cross-sectional
Kim et al. (2010)	LMX theory	South Korea	Five-star hotels	232 non-supervisory employees and 88 supervisory employees	Cross-sectional
Kramer (1995)	LMX theory; assimilation perspective	USA	Government agencies, private companies, and military units	Transferees 91 (Time1), 89 (Time2), 85 (Time3), and 69 (Time 4).	Four-wave panel (1 year)
Lai et al. (2019)	LMX theory	Taiwan	Hospitals	452 full-time nurses	Two-wave panel and interview (3 months apart)
Molines et al. (2022)	Social exchange theory; conservation of resources theory	France	French police force	806 police officers from 35 French police stations	Two-wave panel (8 weeks apart)
Morrow et al. (2005)	LMX theory	USA	A medium-sized trucking firm	207 drivers	Tracking resignations (1 year)
Nelson (2017)	LMX theory; motives of ostracism	USA	A non-profit organization, a for-profit organization (an insurance company)	134 employees	Cross-sectional
Perry et al. (2022)	LMX theory; affective events theory	USA	Study 1: a mid-sized skilled-trade company Study 2: four full-service hotels	Study 1: 145 front-line workers and 41 supervisors Study 2: 266 employees and 41 supervisors	Study 1: cross-sectional Study 2: two-wave panel (1 month after Time 1)
Wang et al. (2023)	LMX theory	China	Chinese companies	167 employees and their matched supervisors	Cross-sectional

**TABLE 2:** Summarized results of articles identified for review.

References	LMX indicator	Outcome	Analysis	Term of LMX (linear)	quadratic term of LMX	% change in R^2^	Other findings
Carnevale et al. (2020)	LMX	Felt obligation for constructive change: FOCC	SEM^1)^	β = 0.30, *p* < 0.01	β = −0.12, *p* < 0.01	7.4%	A negative relationship between LMX and FOCC in the higher levels of co-worker voice or lower levels of leader solicitation of voice.
Golden (2006)	LMX	Job satisfaction	HRA	β = 0.30, *p* < 0.001	β = −0.16, *p* < 0.01	–^1)^	A curvilinear inverted U-shaped relationship between the extent of telecommuting and job satisfaction, mediated by all three relationships (with manager, coworkers, and family).
Harris and Kacmar (2006)	LMX	Stress	HRA	Sample 1: *b* = −1.338, *p* < 0.05 Sample 2: *b* = −0.670, *p* < 0.01	Sample 1: *b* = 0.182, *p* < 0.05 Sample 2: *b* = 0.076, *p* < 0.05	Sample 1: 2.6% Sample 2: 0.6%	LMX cube term did not add any significant variance in either sample.
Harris et al. (2005)	LMX	Turnover intention (ratings of subordinates and leaders)	HRA	Sample 1: *b* = −0.94, *p* < 0.01 Sample 2: *b* = −1.4, *p* < 0.05	Sample 1: *b* = 0.12, *p* < 0.01 Sample 2: *b* = 0.12, *p* < .05	Sample 1: 1.1% Sample 2: 1.9%	The addition of the cubed LMX term was not significant, indicating only one bend in the curve in either sample.
Hesselgreaves and Scholarios (2014)	LMX	Strain (job pressure: JP; lack of organizational support: LOS)	HRA	JP: Junior: β = −0.49, *p* < 0.01; Senior: β = 0.31, ns LOS: Junior: β = −0.44, *p* < 0.01; Senior: β = 0.30, ns	JP: Junior β = 0.73, ns; Senior: β = 2.32, *p* < 0.05 LOS: Junior: β = −0.14, ns; Senior: β = 1.25, ns	JP: Junior 1.0% Senior 7.0% LOS: Junior: 0% Senior: 0%	LMX did not increase stress by raising the demand level for senior subordinates, nor did it have a moderating effect on this relationship.
Hochwarter (2005)	LMX	Job tension	HRA	β = −0.13, *p* < 0.05	β = −0.04, ns	1.0%	For the high negative affection and the low positive affection, the non-linear LMX was significant.
Ionescu and Iliescu (2021)	LMX-MDM	Task performance (ratings of leaders)	MLM	γ = 0.24, *p* < 0.01	γ = −0.06, ns	ns	The LMX–performance relationship was best represented by an inverted U shape for high levels of supervisory interpersonal justice.
Jian (2014)	LMX	Role stressors (ambiguity, conflict, and overload)	HRA	Role ambiguity: β = −0.38, *p* < .001 role conflict: β = −0.31, *p* < 0.001 role overload: β = −0.28, *p* < 0.01	Role ambiguity: β = −0.11, ns role conflict: β = −0.15, *p* < 0.05 role overload: β = −0.21, *p* < 0.01	Role ambiguity: 1.0% role conflict: 2.0% role overload: 3.0%	No evidence for a cubic relationship between LMX and any of the three perceived role stressors.
Kim et al. (2010)	LMX	Turnover intention	HRA	Non-supervisory: β = 0.115, ns Supervisory: β = −0.281, *p* < 0.05	Non-supervisory: β = 0.175, *p* < 0.05 supervisory: β = 0.032, ns	Non-supervisory: 0.1% Supervisory: 2.7%	No evidence for a cubic relationship between LMX and turnover intent in either of the samples.
Kramer (1995)	Trichotomous relationships	Social support and transferees’ adjustment	ANOVA	–	–	–	Middle-group supervisor relationships have the most positive impact of the other two types of relationships (overseer and partnership).
Lai et al. (2019)	LMX	Turnover intention	HRA	β = −0.16, *p* < 0.10	β = 0.03, ns	2.0%	TMX has a U-shaped relationship with turnover intention. Moderate levels of LMX and TMX have the lowest turnover intention.
Molines et al. (2022)	LMX-MDM	Emotional exhaustion	HRA	β = −0.157, *p* < 0.001	β = 0.087, *p* < 0.05	3.0%	When transformational leadership increases from moderate to high, the reciprocation norm induced by LMX leads to exhaustion.
Morrow et al. (2005)	LMX-MDM	Voluntary turnover	HRA	β = −10.974, *p* < 0.05	β = −3.551, *p* < 0.05	-^2)^	The relationship between LMX and turnover is a U-shaped relation, with the possibility that the relationship is S-shaped.
Nelson (2017)	LMX	Ostracism	HRA	β = 0.533, *p* < 0.05	β = −0.068, *p* < 0.01	5.7%	A polynomial (S-shaped) effect existed between LMX and ostracism.
Perry et al. (2022)	SLMX	Safety enforcement	MLM	Study 1: β = –1.95, ns Study 1: β = –1.00, *p* < 0.05	Study 1: β = 0.28, *p* < 0.05 Study 2: β = 0.14, *p* < 0.05	Study 1: 3.0% Study 2: 1.0%	U-shaped relationship between SLMX and safety enforcement emerges at lower safety commitment.
Wang et al. (2023)	LMX	Prohibitive voice	HRA	β = 0.170, *p* < 0.01	β = −0.21, *p* < 0.05	3.0%	No evidence of the moderating effects of regulatory focus on the LMX-prohibitive voice curvilinear relationship.

SEM, structural equation modeling; HRA, hierarchical regression analysis; MLM, multilevel modeling; *b*, unstandardized regression coefficient; β, standardized regression coefficient; γ, fixed-effect coefficient from MLM. These coefficients are reported as presented in the original studies and are not directly comparable.

^1)^This study tested whether the additional variance when adding a quadratic term of leader-member exchange (LMX) to the model was significant after ensuring the curvilinear effect.

^2)^The linear, squared, and cubed LMX terms are added to the same step and analyzed.

### 3.1 Study description

Among the 16 studies we selected, the research that demonstrated the TMGT effect based on the LMX concept was Kramer’s study published in 1995. Kramer’s study classified leader-member relationships into three types and compared members’ sense of adaptability. Subsequent studies quantitatively measured LMX and analyzed its relationship with outcomes such as member stress and turnover intention. Based on publication year, one study (6.3%) appeared in 1995, five studies (31.3%) each were published in the 2000–2009 and 2010–2019 periods, and five studies (31.3%) have already been reported in the 2020–2024 period (the search period for this review).

Based on country of study, nine of the 16 papers (56.3%) were from the United States, three were from Europe (UK, France, and Romania: 18.8%), and four were from Asia (two from China, South Korea, and Taiwan: 25.0%). There was a tendency toward a bias toward Western samples, which exceeded 70% of the total. Regarding the organizations studied, research was conducted on diverse samples including high-technology firms, public organizations, police offices, hotels, and military units. Studies focusing on stress or turnover (intent) particularly collected data from industries with high turnover rates or where increased workload and complexity were concerns (e.g., the hotel industry, hospitals, and the financial services industry).

Furthermore, of the 19 studies included in the 16 reviewed papers, 12 studies (63.2%) employed cross-sectional designs using (self-reported) questionnaires (Table 1). Seven studies (36.8%) used time-lagged or longitudinal designs, and none utilized psychophysiological measures.

The LMX scale used in the literature comprised 12 studies (75.0%). These studies employed the scale developed by Graen and Cashman (1975), Scandura et al. (1986), or the LMX-7 scale developed by Graen et al. (1982), or the slightly modified LMX-7 scale developed by Graen and Uhl-Bien (1995) (including Perry et al., 2022, who adapted it to a supervisor-rated format). Three studies (18.8%) used the LMX-MDM scale developed by Liden and Maslyn (1998). The prevalence of these scales aligns with the trend in previous LMX research examining linear models (Liden et al., 2016; Martin et al., 2016). One study (6.3%) used the trichotomous LMX scale developed by Kramer (1995).

### 3.2 LMX’s TMGT effects

#### 3.2.1 Outcome variables

Let us examine the outcomes examined in studies investigating the TMGT effect of LMX. Following the work of Mumtaz and Rowley (2020), we categorized outcomes into three main types: cognitive/perceptual, affective, and behavioral/attitudinal. We added two additional categories: composite outcomes and others (others’ reactions/actions). Cognitive outcomes were defined as individual thoughts and mindsets. One study (Carnevale et al., 2020) on a sense of obligation to change was classified in this category (6.3%). Two studies by Golden (2006), Kramer (1995), which measured job satisfaction, yielded affective outcomes. However, because Kramer (1995) treated this as part of adjustment alongside leader support provision, stress, and role ambiguity, it was included in the composite outcomes.

Behavioral tendency outcomes included individual behavioral patterns and habits. Six studies measuring turnover (intent) and restrained voice fell into this category (37.5%; four studies measured turnover intent or turnover behavior). Composite outcomes were classified as stress indicators encompassing emotions and cognitive/evaluative aspects, including the aforementioned sense of adjustment (Kramer, 1995) with organizational support, as well as emotional exhaustion states and perceptions of roles (six studies: 37.5%). The remaining two studies (12.5%) did not survey members’ outcomes; instead, they evaluated leaders’ safety-enforcement behaviors or exclusionary behaviors (ostracism) toward other members. Based on the above results, the outcomes demonstrating the TMGT effect of LMX were primarily behavioral/attitudinal tendencies centered on turnover intention and its composite outcome (stress).

#### 3.2.2 Linear effects of LMX and TMGT effects

Only one study failed to fully support the TMGT effect of LMX (Lai et al., 2019). Analysis of data on turnover intentions among hospital nurses revealed only a significant negative association with LMX. The other two studies indicated that the TMGT effect was limited. Ionescu and Iliescu (2021) examined LMX using a four-dimensional LMX-MDM scale. No overall quadratic effect of LMX (combining all four dimensions) on task performance was found. However, the LMX affect, reflecting resource exchange in the emotional dimension, was found to have an inverted-U-shaped effect on task performance. Another study by Jian (2014) examined three indicators of role stress. Role ambiguity showed a negative linear relationship, whereas role conflict and role overload exhibited inverted-U-shaped effects on LMX.

Four studies identified boundary conditions for the TMGT effect of LMX (Hesselgreaves and Scholarios, 2014; Hochwarter, 2005; Ionescu and Iliescu, 2021; Kim et al., 2010), which will be discussed in the next section.

Furthermore, eight of the 19 studies reported data that attempted to analyze cubic curves. Only one of these studies found that a significant percentage of variance increased for the cubed LMX (Nelson, 2017). This study predicted that low-LMX members would be ostracized due to perceived low social skills or competence, whereas high-LMX members would be ostracized for being perceived as exploiters of limited resources who create unfairness. The analysis results showed a somewhat complex curve, suggesting that members with excessively high LMX may experience painful negative consequences. However, the increase in variance from the LMX cubed term was modest, and it is hoped that with methodological improvements, opportunities for further research will increase.

#### 3.2.3 Moderating variables of LMX’s TMGT effect

The reality may be better reflected by the view that the TMGT effect of LMX is not consistently present, but rather a phenomenon observed only under specific conditions influenced by individual differences and contextual/environmental factors. Several studies support this. For example, Hochwarter (2005) found that the relationship between LMX and task performance follows an inverted U-shaped curve only when individuals exhibit high affectivity (or low positive affect). Ionescu and Iliescu (2021) reported that the TMGT effect of LMX (affect) on task performance was best explained when perceptions of leader interpersonal justice were high.

However, findings from studies on moderation effects also suggest that further examination is needed. For instance, results from two studies examining moderation effects based on member job level were not entirely consistent. Hesselgreaves and Scholarios (2014) examined the relationship between LMX and strain (job pressure and lack of organizational support) among hospital nurses in the UK. They found a U-shaped effect of LMX on job pressure only among senior nurses. In contrast, Kim et al. (2010) examined turnover intentions among hotel employees in Seoul, South Korea. Their findings showed that the TMGT effect of LMX was observed among non-managerial members, not among managerial members. These discrepancies in the results suggest multiple possible explanations, including differences in job type and the accompanying member autonomy and role ambiguity, differences in outcome type, or differences in cultural context.

### 3.2.4 Mechanism of LMX’s TMGT effect

Several studies have empirically examined the mechanism by which LMX generates the TMGT effect. Hesselgreaves and Scholarios (2014) hypothesized that high LMX creates high demands leading to stress. Unfortunately, this hypothesis was not supported. Similarly, Jian (2014) found that both low and high LMX were associated with lower perceived role demands than moderate LMX. Regarding these results, he offers an interpretation from a socioemotional exchange perspective. Jian (2014) suggests that as LMX quality develops from a low to a certain level, greater socioemotional exchange is promoted, thereby creating unclear obligations and role expectations that increase stress. Beyond this point, as LMX improves, its symbolic meaning becomes clearer, reducing role stress.

Perry et al. (2022) considered the interaction to function as an emotional event. Leaders increase safety-monitoring behavior when reflecting on negative experiences or emotions associated with low LMX relationships, or when demonstrating trust and care toward members with high LMX. A key perspective of this study is that the TMGT effect observed in leaders’ behavior may be mediated by the emotional attitude component of favorability toward members. These findings are noteworthy, as they suggest the involvement of socio-emotional factors in the psychological mechanisms underlying the TMGT effect of LMX, amid a body of research showing that LMX mitigates negative effects (Huang et al., 2018; Park and Nawakitphaitoon, 2018; Wu et al., 2024).

## 4 Proposal of an integrated model

Given this overview, a growing body of evidence suggests that LMX can ironically weaken some of the outcomes it is expected to enhance. Here, we connect the observed occurrence of the TMGT effect in LMX with its theoretical underpinnings. The emergence of the TMGT effect can be predicted based on LMX theory, specifically from the perspective of (the fear of) resource depletion relative to job demands. However, resource scarcity alone cannot fully explain why excessive LMX leads to undesirable outcomes, such as suppressing the felt obligation to pursue constructive change, inhibiting voice among high LMX members, or their exclusion by other members. Therefore, this paper fills this gap by drawing on prospect, organizational justice and self-determination theories to propose an integrated theoretical framework.

According to LMX theory, the development of the quality of leader-member relationships depends significantly on the exchange of economic and socio-emotional resources (Jian, 2014). Integrating this with prospect theory (Kahneman and Tversky, 1979) may help unravel the mechanism behind the TMGT effect. High LMX individuals reference their already abundant resources as a baseline and perceive the privileged relationship, supported by a leader’s trust and respect, as a valuable benefit worth protecting. Beyond a certain threshold, this perception diminishes the satisfaction derived from further gains. Simultaneously, it fosters excessive vigilance, anxiety, and negative emotions such as fear of losing face or status. Even minor losses cause significant distress and trigger shame over losing a leader’s trust (Xing et al., 2021).

Confronting this internal distress and conflict accelerates stress (Danauskë et al., 2023; Lok and Bishop, 1999) or leads to attempts to avoid it by suppressing dissent toward the leader or engaging in impression management (Bryant and Merritt, 2021; Carnevale et al., 2020). This explains the tendency among high LMX individuals to suppress their felt obligation for constructive change and a prohibitive voice.

Regarding the psychology and behaviors that generate exclusion of high LMX individuals, even when reinforced by prospect theory, ambiguities remain. As LMX theory posits, leaders are categorized into in-groups and out-groups as they build diverse relationships, and the quality of LMX differs between these groups. This LMX quality becomes a subject of social comparison (Blader and Tyler, 2009; Smith et al., 2012). For low LMX individuals, high LMX members are perceived as sources of workplace unfairness (Choi et al., 2020; Martin et al., 2016), making them prone to negative emotions, such as envy and anger (Greenberg et al., 2007; Lee et al., 2022; Nelson, 2017; Tremblay et al., 2021). Consequently, individuals with high LMX find it difficult to obtain useful resources from colleagues and are instead exposed to aggressive behavior by low LMX individuals (Beshai et al., 2022; Pan et al., 2021; Reh et al., 2018).

Another crucial perspective concerns the extent to which individuals act with initiative, will, and choice (i.e., the perspective of autonomous motivation). Deci et al. (2017) emphasized the importance of workplace autonomy as a source of intrinsic motivation, arguing that external regulation, such as a leader’s management style, influences this aspect. Shifting our focus back to high LMX individuals through this lens, they should, in principle, benefit from autonomy within a leader’s resources and enhanced support, thereby becoming intrinsically motivated. However, the abundance of resources strengthens socio-emotional bonds, such as a sense of indebtedness or gratitude toward a leader, personal trust, and loyalty, which then introduce a different quality and complexity.

Social-emotional resources are abstract rather than concrete, and the more this exchange is encouraged, the greater members’ dependence on the leaders who provide them (Cropanzano et al., 2017; Jian, 2014). This heightened dependence deprives members of opportunities to solve problems themselves, hinders the fulfillment of competence, and creates interpersonal environments prone to negative spirals. Additionally, hostility and envy from other members stemming from workplace LMX imbalance constitute an unpleasant experience for those with high LMX (Lee et al., 2018). These interactions with leaders and other members represent external controlling pressures for high LMX individuals. This interpersonal pressure undermines the quality of their autonomous motivation, likely leading them to refrain from proactive behavior.

In summary, the TMGT effect of LMX is thought to occur when the quality of LMX exceeds a threshold, and the resulting resource scarcity and associated psychological strain, particularly the socio-emotional costs, outweigh the gains derived from the high-quality relationship. The JD-R model, COR, and cognitive load theory explain this by positing that the depletion (or fear of depletion) of available resources relative to job demands generates psychological strain. The potential for exacerbating existing resource depletion alongside socio-emotional costs can be derived both from the perspective of privileged relationships explained by prospect theory and from the perspective of boundaries and psychological distance from other members, as posited by organizational justice theory. Beyond these perspectives of resource loss, self-determination theory reinforces the TMGT phenomenon from a motivational standpoint. Through this process involving resources—emotions—motivation (REM), it can be said that stress induction and the inhibition of proactive behavior occur.

## 5 Discussion

### 5.1 Summary of results

This review reexamines the LMX paradigm from the perspective of the effect of “too much of a good thing.” Although LMX has traditionally been positioned as a positive relationship-building process, integrating empirical evidence and theoretical insights reveals a paradox: the simultaneous presence of abundant resources and psychological costs. From a theoretical perspective, the psychological costs of high LMX are not merely hypothetical but are rooted in LMX theory. Members of high LMX relationships, characterized by trust, obligation, and respect, may incur hidden costs through the emotional labor required to maintain their privileged connection. We propose that this arises from a perceived scarcity of supportive resources and a violation of autonomous motivation, suggesting a theoretical framework for TMGT encompassing the REM model.

### 5.2 Limitations and directions for future research

This review presented novel and integrated perspectives on the TMGT effect of LMX, but it would be premature to conclude that this phenomenon exists. Our understanding of the variables associated with this specific effect also remains limited. To contextualize these findings, several limitations must be acknowledged. Therefore, we propose themes focusing on the following three primary areas for future research.

First, although this review suggests an emotional dimension underlying the TMGT effect of LMX, the mechanisms remain poorly supported empirically. Few studies have explicitly demonstrated the non-linear effects of LMX, and even fewer have examined mediating or moderating variables. It is necessary to examine the range of outcomes where the TMGT effect is observed. Behavioral-level outcomes are particularly important as they directly impact organizational performance. The three interactions (resource depletion, negative emotions, and suppression of autonomy) are expected to inhibit the exercise of voice and creativity, which require higher-order competencies, while promoting ingratiation toward leaders.

Regarding mechanisms, based on the REM research framework, efforts to elucidate and model mediating factors related to emotional exhaustion and social comparison are anticipated. Regarding moderators, factors such as industry/job type (Lai et al., 2019) and competitive workplace environments are conceivable. High LMX individuals with a sense of entitlement may leverage a leader’s trust to expand their power in competitive workplaces, potentially leading to self-serving behaviors and norm violations. Additionally, individual attachment styles (Bowlby, 1969, 1973; Mikulincer and Shaver, 2008) and contextual/cultural factors are anticipated. Members with insecure attachment tendencies, who strongly desire to maintain a high LMX relationship, or those in collectivist or high-power-distance cultures, are expected to experience heightened psychological strain due to dependency on leader approval and efforts to sustain the relationship.

Furthermore, as the nature of work continues to evolve, the conceptualization of LMX may also change. For instance, is establishing high-quality LMX through digital means alone even possible? Can the TMGT effect of LMX be realized in virtual environments? If there are inherent limitations to digital means in relationship building, this effect may not occur. Future research is expected to deepen our understanding of LMX’s characteristics and phenomena, including perspectives on the use of digital means.

Second, LMX inherently involves socio-emotional costs. To develop leadership practices that maximize relational benefits while minimizing unintended consequences, it is essential to unravel this emotional involvement. However, the majority of the reviewed studies rely on cross-sectional self-report surveys, which have limitations in capturing the dynamic nature of emotions and temporal causality. Gooty et al. (2019) argue for the need to collect and analyze longitudinal data combining subjective and objective measures, moving beyond methodological limitations such as common method variance and cross-sectional surveys.

Nelson (2017), analyzing the relationship between LMX and ostracism, noted that although respondents meeting high-quality, high-isolation criteria were few, this dynamic remains a critical factor for organizations. This observation is also significant as it raises an analytical issue: the small number of respondents in this category may prevent the observation of a clear positive correlation. Given this point, addressing the dynamic nature of social-emotional states requires research designs and measurement methods capable of capturing subtle changes across the temporal and unconscious dimensions of an individual’s psychological burden. We recommend shifting toward a multimodal, longitudinal, context-sensitive approach incorporating experiential sampling, psychophysiological monitoring, and behavioral trace data. A comprehensive evaluation that combines multiple indicators (Table 3), selected optimally for specific objectives and research/experimental contexts, will enhance the validity of the results and interpretations.

**TABLE 3:** Longitudinal measurement indicators and application examples in leader-member exchange (LMX) interactions.

Indicators	Definition	Primary measurement targets	Usage examples
Measurement of subjective self-reports based on experience			
Experience sampling method (ESM)	Intra-individual variation at multiple points in time throughout the day	Psychological, behavioral, or physical aspects such as emotions, stress, and relationship awareness	Quality of leadership-member exchange (e.g., in which situations and what kind of support is being provided by leaders) in relation to everyday workplace events
Day reconstruction method (DRM)	Recalling and reporting the events of the previous day		
Work day reconstruction method (W-DRM)			
Measurement of objective physiological responses			
Heart rate variability (HRV)	Variation in time intervals between heartbeats	Autonomic nervous system activity	Stress responses to feedback from leaders
Electrodermal activity (EDA)	Electrical conductance of the skin influenced by sweat gland activity	Sympathetic nervous system response	Emotional reactions and arousal levels when reprimanded by a leader
Electroencephalography (EEG)	Electrical activity of the brain measured via scalp electrodes	Brainwave patterns, cognitive states	Measuring attentional focus, cognitive load in communication, and fatigue detection
Functional near-infrared spectroscopy (fNIRS)	Brain hemodynamics via light absorption differences	Oxygenated/deoxygenated hemoglobin levels	Visualization of cognitive load in response to leader instructions, and brain activity related to comprehension and judgment
Facial thermography	Infrared imaging of facial temperature distribution	Peripheral blood flow, emotional response	Detecting stress among subordinates, suppressing their emotions through facial temperature changes
Passive and non-invasive monitoring of physiological responses			
Wearable devices	Sensors embedded in wearable technology	Heart rate, movement, sloop, skin conductance	Monitoring heart rate, activity, and the effects of sleep deprivation during meetings
Recording of behavior log			
Digital trace data	Behavioral logs from digital interactions	Behavioral and cognitive patterns	Analyzing delays in email responses and changes in typing speed

By examining multimodal research incorporating behavioral observation and physiological measurement, we can verify the emergence of socio-biological synchrony between leaders and members (Chauvigné et al., 2019; Mayo and Gordon, 2020; Wass et al., 2020). We can also elucidate mechanisms underlying the emergence of socio-emotional costs in leader-member pairs, as well as stress accumulation and diminished behavioral performance. Constructing a multi-layered assessment within leader-member interactions would enable a deeper understanding of the dynamic interplay between psychological and physical dimensions.

Third, the literature examined in this review was subject to publication bias and sample diversity constraints. While identifying key trends and findings regarding LMX effects, reliance on cross-sectional studies meant that conclusions were inherently influenced by the methodological choices made in the included studies. Furthermore, the inclusion criteria focused on studies attempting to empirically test hypotheses about the TMGT effect of LMX. Consequently, studies demonstrating indirect associations that could moderate non-linear effects, the TMGT effect via LMX differentiation (Lee and Chae, 2017; Sui et al., 2016), or implicit non-linear patterns were excluded. Future research requires accumulating more evidence and conducting broader and multifaceted analyses.

### 5.3 Practical implications

Recognizing the potential drawbacks of excessively high-quality LMX relationships offers important implications for organizational leadership, human resource management, and psychological safety in the workplace. Demonstrating care for individuals while fostering mutual trust is a hallmark of effective leadership in the workplace. However, this review emphasizes that achieving sustainability requires balancing psychological intimacy with resources, specifically, intrinsic motivation. Given this importance, an interdisciplinary approach incorporating organizational behavior, neuroscience, and human-centered technology holds promise not only for academic interest but also for practical application across these fields.

Organizations may need to refrain from assuming that “high-quality LMX always leads to favorable outcomes.” In such cases, human resource (HR) professionals and managers must implement leadership training and boundary management of job roles and responsibilities to heighten awareness of the potential risks associated with emotionally involved management. This offers important implications for organizational practice and leader development. Although fostering trust and mutual respect underpine an organization, leaders must be mindful of the psychological burden imposed on members through excessive involvement or unrealistic expectations. By recognizing the duality of LMX, practitioners within organizations, particularly HR departments, can design more sophisticated strategies to enhance leadership effectiveness and member well-being. One such strategy is leadership development. Another is HR maintenance practices utilizing AI and monitoring systems.

Leadership development programs should incorporate training modules that promote awareness of emotional regulation and relational asymmetry. Leaders can contribute to creating appropriate workplace environments by cultivating the ability to recognize signs of emotional over-involvement, such as micromanagement or chronic over-support, that unintentionally increase members’ psychological burdens. Alternatively, efforts are underway to use animation technology to map real-time heart rate fluctuations to flame intensity (Wang and Okada, 2023). Adjusting one’s reactions in real-time based on changes in the other person’s flame is expected to be a unique tool for building positive relationships and facilitating dialogue, as well as a useful intervention for coaching.

Recent advances in AI-powered communication analysis, emotion detection, and digital coaching suggest new pathways for enhancing LMX monitoring in remote environments. Although reliance on such tools raises ethical and privacy concerns, highlighting the need for responsible management and integration, certain benefits can be anticipated. The TMGT phenomenon suggests that regular recovery, flexibility, and moderation are essential for sustaining long-term functionality. Therefore, timely identification and response to instances where LMX strength becomes unbalanced with job roles or organizational goals will contribute to optimizing team relationships.

## 6 Conclusion

The existence of the TMGT effect provides a powerful lens for understanding this paradox. A review of this phenomenon suggests that although high-quality LMX relationships are generally associated with desirable outcomes, excessively high LMX may induce unintended psychological side effects. This paper contributes to a more balanced understanding of LMX dynamics by assessing methodological limitations in elucidating psychological mechanisms and proposing new research directions.

We propose leadership development that recognizes the complexity and potential risks inherent in relational excellence. Future research integrating longitudinal designs, psychophysiological assessments, and practical interventions, while examining both member and leader perspectives, will yield richer insights into LMX effects and mechanisms. Furthermore, understanding future trends in LMX necessitates interdisciplinary research spanning psychology, organizational behavior, information systems, and ethics. This will provide researchers and practitioners with insights into fostering healthier and more sustainable leader-member relationships. Consequently, it is expected to promote the development of educational programs and intervention methods that cultivate appropriate coping skills without undue influence.
